# Application of *QPLEX*^*TM*^ biomarkers in cognitively normal individuals across a broad age range and diverse regions with cerebral amyloid deposition

**DOI:** 10.1038/s12276-021-00719-3

**Published:** 2022-01-20

**Authors:** Dongjoon Lee, Jong-Chan Park, Keum Sim Jung, Jiyeong Kim, Ji Sung Jang, Sunghoon Kwon, Min Soo Byun, Dahyun Yi, Gihwan Byeon, Gijung Jung, Yu Kyeong Kim, Dong Young Lee, Sun-Ho Han, Inhee Mook-Jung

**Affiliations:** 1 Department of Biochemistry and Biomedical Sciences, College of Medicine, Seoul National University, Seoul, 03080 Republic of Korea; 2 SNU Dementia Research Center, College of Medicine, Seoul National University, Seoul, 03080 Republic of Korea; 3 Neuroscience Research Institute, Medical Research Center, College of Medicine, Seoul National University, Seoul, 03080 Republic of Korea; 4 Department of Neurodegenerative Disease, UCL Queen Square Institute of Neurology, University College London, London, WC1E 6BT UK; 5 QuantaMatrix Inc, Seoul, 03080 Republic of Korea; 6 Department of Psychiatry, Pusan National University Yangsan Hospital, Yangsan, 50612 Republic of Korea; 7 Department of Neuropsychiatry, Seoul National University Hospital, Seoul, 03080 Republic of Korea; 8 Department of Nuclear Medicine, SMG-SNU Boramae Medical Center, Seoul, 07061 Republic of Korea; 9 Department of Psychiatry, College of Medicine, Seoul National University, Seoul, 03080 Republic of Korea; 10 Institute of Human Behavioral Medicine, Medical Research Center, Seoul National University, Seoul, 03080 Republic of Korea

**Keywords:** Alzheimer's disease, Neural ageing, ELISA

## Abstract

The deposition of beta-amyloid (Aβ) in the brain precedes the onset of symptoms such as cognitive impairment in Alzheimer’s disease (AD); therefore, the early detection of Aβ accumulation is crucial. We previously reported the applicability of the *QPLEX*^*TM*^ Alz plus assay kit for the prescreening of Aβ accumulation. Here, we tested the specific application of the kit in a large cohort of cognitively normal (CN) individuals of varying ages for the early detection of Aβ accumulation. We included a total of 221 CN participants with or without brain Aβ. The *QPLEX*^*TM*^ biomarkers were characterized based on age groups (1^st^–3^rd^ tertile) and across various brain regions with cerebral amyloid deposition. The 3^rd^ tertile group (>65 years) was found to be the most suitable age group for the application of our assay kit. Receiver operating characteristic curve analysis showed that the area under the curve (AUC, discrimination power) was 0.878 with 69.7% sensitivity and 98.4% specificity in the 3^rd^ tertile group. Additionally, specific correlations between biomarkers and cerebral amyloid deposition in four different brain regions revealed an overall correlation with general amyloid deposition, consistent with previous findings. Furthermore, the combinational panel with plasma Aβ1–42 levels maximized the discrimination efficiency and achieved an AUC of 0.921 with 95.7% sensitivity and 67.3% specificity. Thus, we suggest that the *QPLEX*^*TM*^ Alz plus assay is useful for prescreening brain Aβ levels in CN individuals, especially those aged >65 years, to prevent disease progression via the early detection of disease initiation.

## Alzheimer’s disease: a blood test may offer early diagnosis

A novel assay kit called *QPLEX*^*TM*^ Alz plus assay offers a convenient method for assessing brain levels of the beta-amyloid proteins implicated in Alzheimer’s disease in people with normal cognitive abilities, especially those aged over 65. South Korean researchers led by Inhee Mook-Jung at Seoul National University assessed the efficacy of blood tests using the *QPLEX*^*TM*^ kit on 221 participants in the Korean Brain Aging Study for Early Diagnosis and Prediction of Alzheimer’s Disease (KBASE). The researchers developed the assay to identify several circulating biomarkers of brain beta-amyloid accumulation. They found the test can distinguish between people known to either have or not have beta-amyloid deposits in their brain. This suggests *QPLEX*^*TM*^ Alz plus assay could offer an improved procedure for easy and early diagnosis of Alzheimer’s, increasing the opportunities for effective early treatment.

## Introduction

Alzheimer’s disease (AD) is the most prevalent form of the disease that causes dementia, affecting more than 40 million individuals worldwide^1^. There are various causes of Alzheimer’s disease^2–4^, and the primary pathology of AD comprises severe amyloid deposition and neurofibrillary tangles in the brain^5^. Multiple methods for assessing brain amyloid levels have been developed in recent years, including the use of Pittsburgh compound B positron emission tomography (PiB-PET)^6^. However, due to the limited accessibility of PiB-PET, ongoing trials are being conducted to find surrogates for PiB-PET, such as blood biomarkers that reliably indicate brain amyloid levels^7^. Without the need for burdensome cerebrospinal fluid extraction or high-cost brain imaging, blood-based diagnoses have numerous advantages that have drawn the attention of researchers, clinicians, and the elderly population.

Several studies have reported changes in blood biomarker levels during the onset and progression of AD^8–10^. Moreover, the fact that brain amyloid deposition precedes cognitive abnormalities emphasizes the need for screening in cognitively normal (CN) individuals^9^. During the preclinical or presymptomatic stage of AD, PiB-PET–positive individuals, regardless of their cognitive state, are at a risk of developing significant cognitive impairment; this period is considered the optimal treatment period^11^. Considering this, the prediction of AD progression based on blood biomarkers could be beneficial to CN individuals prone to progression toward mild cognitive impairment (MCI) by providing preventive measures and aiding in early intervention.

In our previous studies, we identified a novel blood-based biomarker panel (beta-amyloid 1-40, Aβ1−40; angiotensin-converting enzyme, ACE; periostin, POSTN; and galectin-3 binding protein, LGALS3BP) to predict cerebral amyloid deposition^11,12^. Evidence for the relevance of Aβ1−40 and ACE to AD has been reported previously^13–16^. Aβ1−40 is one of the most canonical forms of an AD blood biomarker^13^. ACE is known to directly degrade beta-amyloid protein^14^, retard beta-amyloid aggregation^15^, and convert Aβ1−42 to Aβ1−40^16^. In the case of POSTN and LGALS3BP, their roles in the immune response suggest possible relevance to AD pathology. POSTN can recruit eosinophils^17^; LGALS3BP is known to be upregulated in activated microglia^18^ and plays a role in inflammation in the periphery through inhibition of neutrophil activation upon binding of its ligand Gal-3^19^ and modulation of natural killer cell activity and interleukin production^20^.

We developed a prescreening platform for PiB-PET positivity using this *QPLEX*^*TM*^ panel for individuals >55 years old, including CN individuals and patients with MCI and dementia^21^. Here, we investigated the application of the *QPLEX*^*TM*^ Alz plus assay kit to CN individuals for the early diagnosis of AD and aimed to demonstrate the applicability of this kit in distinguishing between CN individuals with and without amyloid burden, especially in restricted age groups (the 3^rd^ tertile group, >65 years), for an efficient diagnosis. The additional use of plasma beta-amyloid (Aβ) 1−42 with respect to the biomarkers currently in the kit was expected to improve diagnostic performance. Thus, we aimed to investigate whether the *QPLEX*^*TM*^ Alz plus assay kit is a useful method for detecting and prescreening cerebral amyloid deposition in preclinical AD.

## Materials and methods

### Participants

A total of 221 CN individuals were included in this cross-sectional study. All participants were recruited as part of the Korean Brain Aging Study for Early Diagnosis and Prediction of Alzheimer’s Disease (KBASE) and underwent appropriate clinical and neuropsychological assessments in accordance with the KBASE assessment protocol. The recruitment of participants, clinical diagnostic criteria for AD, and further information are detailed in our previous reports involving the same KBASE cohort^11,21,22^.

### PiB-PET

All participants underwent PiB-PET scans using a 3.0-Tesla PET-magnetic resonance (MR) scanner (Siemens Healthineers, Erlangen, Germany). Each participant was intravenously injected with 555 MBq of [11 C] PiB (450–610 MBq) PET tracer, which enabled the visualization of cerebral amyloid deposition. The automatic anatomic algorithm determined the degree of amyloid accumulation, which was calculated using the standardized uptake value ratio (SUVR). Four regions of interest (ROIs) in the brain were chosen within the following regions: the lateral temporal (LT), lateral parietal (LP), posterior cingulate-precuneus (PC-PRC), and frontal (FR) regions. These regions were identified by an automatic anatomic algorithm and a region-combinative method, as reported previously^23^. We determined the negative and positive cutoff values for PiB-PET based on the Mayo Clinic Alzheimer’s Disease Research Center and Mayo Clinic Study of Aging criteria using the same approach as that described previously^24–28^. If the SUVR value was ≥1.4 for at least one of the four ROIs, the individual was defined as PiB-positive (PiB^+^). Additional information on the imaging protocols was provided in our previous study^12,22^.

### Blood sampling

All fasting blood samples were collected at 9:00 am. Whole blood samples were collected in K2 EDTA tubes (BD Vacutainer Systems, Plymouth, UK) and centrifuged at 700 × g for 5 min at room temperature (RT). The supernatant was collected and centrifuged, and the tubes were stored at −80 °C.

### xMAP technology for the quantification of plasma Aβ1–42 and Aβ1–40

To quantify the concentrations of plasma Aβ1–42 and Aβ1–40, we performed a Bioplex 200 assay (Bio–Rad, Hercules, CA, USA) using xMAP technology and an INNO-BIA plasma Aβ forms kit (Fujirebio Diagnostics, Gothenburg, Sweden) in accordance with the manufacturer’s guidelines^12^. Briefly, plasma samples were diluted 3-fold in plasma diluent buffer. After washing the plate to initialize the filter, the diluted beads were added to the wells of the plates and transferred to the filters. Next, the conjugated beads, standards, samples, and controls were added and incubated overnight. The next day, each well was washed and incubated with the detection solution for 1 h. After incubation, further washing steps were performed, and the reading solution was added. Finally, the levels of plasma Aβ1–42 and Aβ1–40 were quantified using the Bioplex 200 system.

### QPLEX^TM^ Alz plus assay

All quantification methods were detailed in our previous study^21^. Briefly, the *QPLEX*^*TM*^ kit utilized Quantamatrix’s multiplex diagnostics platform (QMAP; Quantamatrix Inc., Seoul, Republic of Korea)^29^. First, human plasma samples (singular) were diluted in diluent buffer and incubated with the coded beads and biotin-conjugated detection antibodies (angiotensin-converting enzyme, ACE, DY929, R&D Systems, Minneapolis, USA; galectin-3 binding protein, LGALS3BP, DY2226, R&D Systems; periostin, POSTN, DY3548B, R&D Systems; Aβ1−40, 014-26923, Wako, Japan). The immunocomplexes were washed twice with washing buffer at a Biotek-510 magnetic wash station (Biotek, VT, USA). Diluted R-phycoerythrin-conjugated streptavidin was added to each well. After three washes, the immunocomplexes were resuspended in 100 μl of washing buffer by tapping and automatically analyzed using the QMAP^TM^ system. The intra/interassay coefficients of variation and limits of detection were as follows: ACE, intra: 4.9%, inter: 5.1%, 0.22 ng/ml; LGALS3BP, intra: 1.1%, inter: 5.7%, 0.04 ng/ml; POSTN, intra: 9.0%, inter: 6.0%, 0.034 ng/ml; and Aβ1−40, intra: 5.3%, inter: 3.9%, 0.50 pM.

### Monotone regression spline analysis

Analyses for monotone penalized regression splines were performed to identify the relationship between each biomarker response and imaging biomarkers^30^. Monotone curves were generated using the smoothing spline method with four knots. To effectively compare different *QPLEX*^*TM*^ biomarkers, their levels were transformed to z scores. The participants’ age acted as a proxy for the progression time.

### Statistical analyses

GraphPad Prism 8 (San Diego, CA, USA) and MedCalc version 20.009 software (Ostend, Belgium) were used for all statistical analyses. Comparison analyses between two variables were conducted by independent *t* tests or analyses of covariance (ANCOVAs) with correction for age and sex. Spearman correlation analysis or partial correlation analysis (with the correction of covariates) was performed as appropriate. Logistic regression analysis followed by receiver operating characteristic (ROC) curve analysis was conducted to determine the discriminatory power, sensitivity, and specificity for each of the biomarker panels. The formulas, coefficients, and constants could be optimized since there were appropriate outliers and various logistic regression models. By using the values of variance inflation factors, multicollinearities were checked. All statistical outliers (two samples for Aβ1–40, three samples for POSTN, four samples for ACE, and no outliers for LGALS3BP) were excluded from the cohort in accordance with Grubb’s double-sided outlier test (*p* < 0.05).

## Results

### Experimental design and categorization of participants

**Fig. 1:**
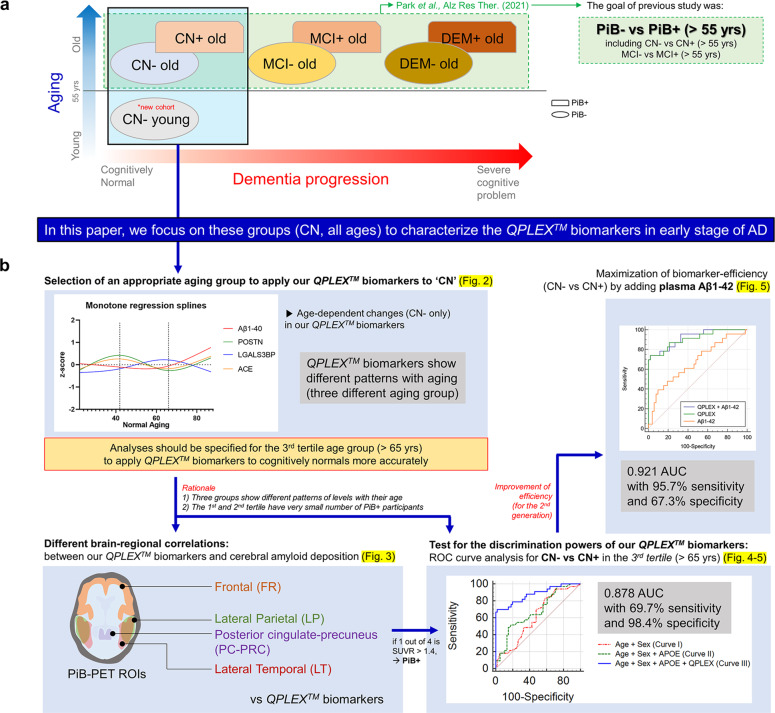
Experimental design. **a** Classification of participants and different brain areas from our previous study. The current study focused on the CN group (including CN^−^ individuals <55 years old), whereas our previous study covered all age-matched cognitive groups with individuals >55 years old. **b** Overall experimental design. The experimental processes were as follows: age-dependent characterization of *QPLEX*^*TM*^ biomarkers, partial correlation analysis between the biomarkers and cerebral amyloid deposition in specific age groups, testing for the discrimination powers of our *QPLEX*^*TM*^ biomarkers in the specific age group, and maximization of biomarker efficiency for CN^−^ vs. CN^+^ with an additional variable.

**Fig. 2:**
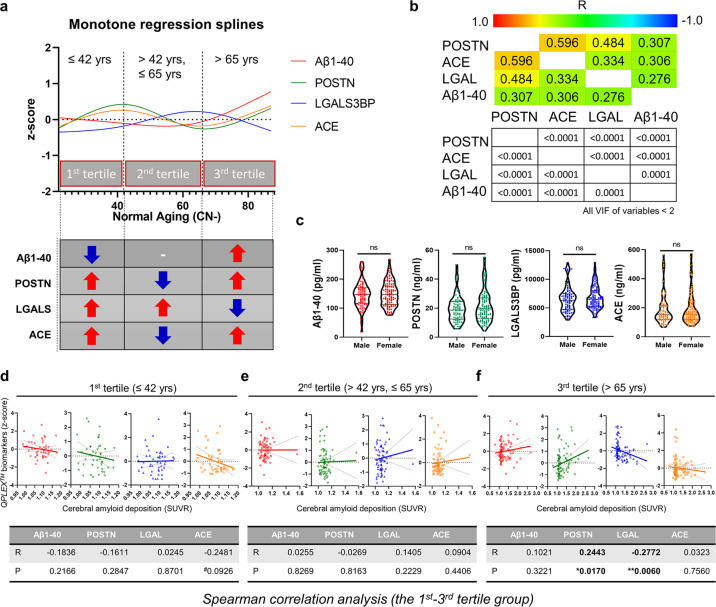
Age-dependent characterization of *QPLEX*^*TM*^ biomarkers in cognitively normal (CN) participants (2a-2c, CN^−^ only; 2d-2f, entire CN group). **a** A monotone spline model for *QPLEX*^*TM*^ biomarkers. The ages of the participants acted as a proxy for time. The levels of *QPLEX*^*TM*^ biomarkers were transformed to *z* scores. The patterns of each biomarker revealed differences based on age groups (the 1st tertile, ≤42 years; 2nd tertile, 42–65 years; 3rd tertile, >65 years). The curves were created by a smoothing spline with four knots. **b** Pearson’s correlation between the *QPLEX*^*TM*^ biomarkers. Colored and white boxes show *R* and *P* values, respectively. We confirmed that all the variables had low values of variance inflation factors (VIF < 2). **c** No significant differences in *QPLEX*^*TM*^ biomarkers between males and females. *P* values were obtained from a two-sided unpaired *t* test. **d**–**f** Correlation between the *QPLEX*^*TM*^ biomarkers and cerebral amyloid deposition (SUVR) based on age group (**d**, 1st tertile; **e**, 2nd tertile; **f**, 3rd tertile). The levels of *QPLEX*^*TM*^ biomarkers were transformed to *z* scores. Only the 3rd tertile group showed significant results. *P* values were obtained from Spearman correlation analysis (cutoff, *p < 0.05, and **p < 0.01). Abbreviations: Aβ1–40 beta-amyloid 1–40, POSTN periostin, LGALS3BP (LGAL) galectin-3 binding protein, ACE angiotensin-converting enzyme, SUVR standardized uptake value ratio.

**Fig. 3:**
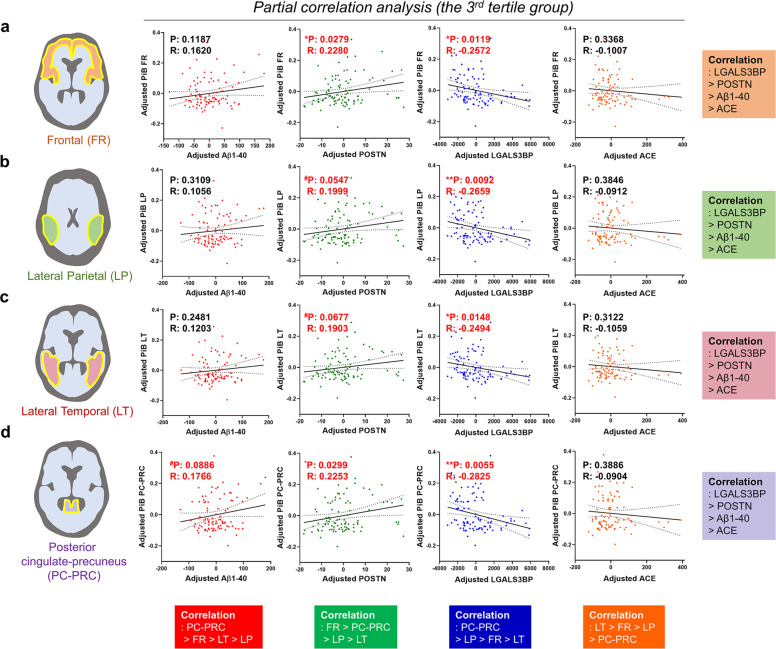
Correlations between the *QPLEX*^*TM*^ biomarkers and cerebral amyloid deposition in various brain ROIs in the 3rd tertile group (>65 years). **a**–**d** Partial correlation analysis between *QPLEX*^*TM*^ biomarkers and cerebral amyloid deposition (SUVR) from four ROIs in the 3rd tertile group (>65 years) with correction for age and sex. *P* value cutoff, ^#^*p* < 0.10, *p < 0.05, and **p < 0.01. P partial correlation *P* value, R partial correlation coefficient. Dotted lines indicate 95% confidence intervals. Abbreviations: ROI region of interest, CN cognitively normal, - or + PiB positivity, Aβ1–40 beta-amyloid 1–40, POSTN periostin, LGALS3BP galectin-3 binding protein, ACE angiotensin-converting enzyme.

**Fig. 4:**
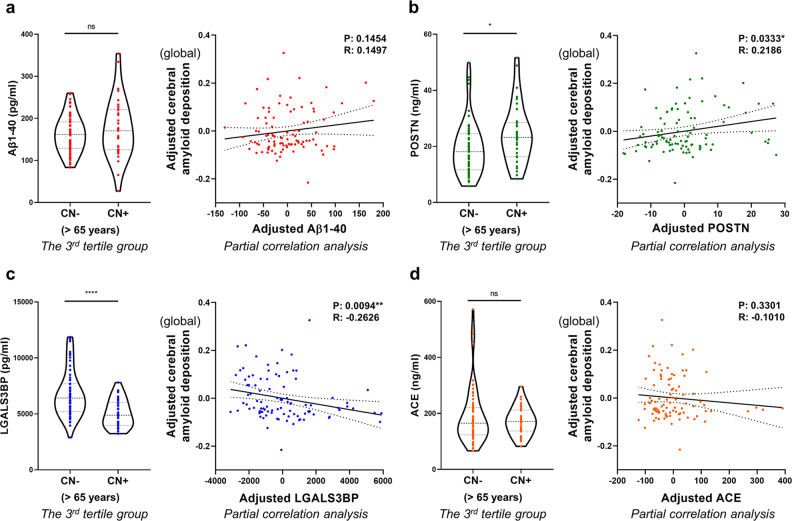
*QPLEX*^*TM*^ biomarkers and cerebral amyloid deposition in the 3^rd^ tertile group (>65 years). **a**–**d** Each graph on the left shows the comparison of *QPLEX*^*TM*^ biomarker levels between CN^−^ individuals and CN^+^ individuals within the 3^rd^ tertile group. A two-sided unpaired *t* test was performed. Each graph on the right shows the results of a partial correlation analysis with correction for age and sex. *P* value cutoff, *p < 0.05, **p < 0.01, ***p < 0.001, and ****p < 0.0001. P partial correlation P value, R partial correlation coefficient. Dotted lines indicate 95% confidence intervals. Abbreviations: CN cognitively normal; − or + PiB positivity, Aβ1–40 beta-amyloid 1–40, POSTN periostin, LGALS3BP galectin-3 binding protein, ACE angiotensin-converting enzyme.

**Fig. 5:**
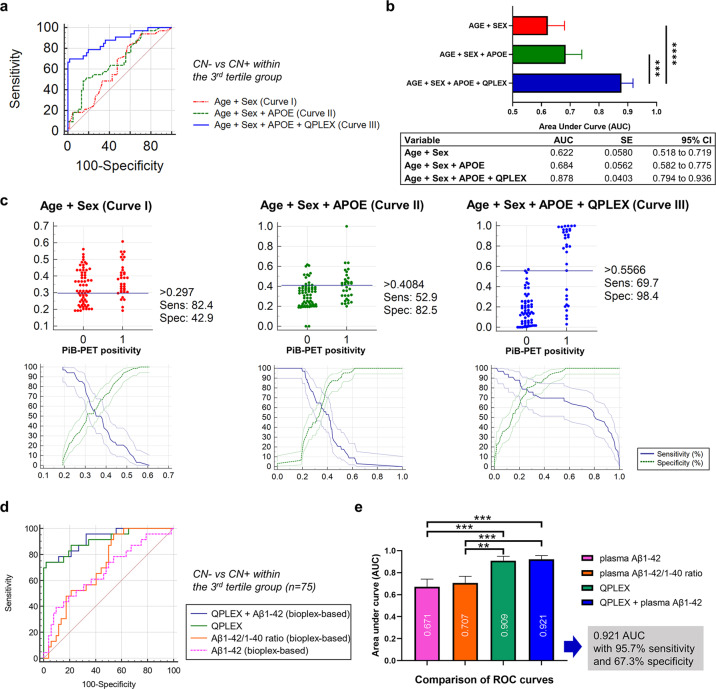
Receiver operating characteristic (ROC) curve analysis for PiB-PET positivity within the 3^rd^ tertile group (>65 years). **a** ROC curve analysis for cognitively normal individuals grouped according to PiB-PET positivity. **b** Comparison of ROC curves with different combinations of age, sex, APOE genotype, and QPLEX^TM^ biomarkers as variables. P values were obtained from Delong’s test (*p < 0.05, **p < 0.01, and ***p < 0.001). **c** Interactive dot diagram (upper) and graph of sensitivity, specificity, and their 95% confidence intervals plotted against the criterion values (lower). **d**,**e** Maximization of the discrimination power for CN^−^ vs. CN^+^. The AUC reached 0.921 with 95.7% sensitivity and 67.3% specificity. P values were obtained from Delong’s test (*p < 0.05, **p < 0.01, and ***p < 0.001). Abbreviations: PiB-PET Pittsburgh compound B positron emission tomography, APOE apolipoprotein E, AUC area under the curve, SE standard error, CI confidence interval.

The overall experimental design of the study is shown in Fig. 1a, b. We previously reported that our *QPLEX*^*TM*^ biomarkers (Aβ1–40, POSTN, LGALS3BP, and ACE) showed significant differences between age-matched (age >55 years old) PiB^−^ and PiB^+^ participants (Fig. 1a)^21^. In this study, we utilized biomarkers to determine whether amyloid accumulated within the CN group (CN^−^, *n* = 185, PiB-PET negative; CN^+^, *n* = 36, PiB-PET positive; Table 1) (Fig. 1a). Notably, we included participants <55 years of age. However, we needed to first identify the age group that was the most suitable CN group for the application of our *QPLEX*^*TM*^ Alz plus assay kit (Fig. 1b). We attempted to identify age-dependent changes in our *QPLEX*^*TM*^ biomarkers and finally concluded that the analyses should be specified for the 3^rd^ tertile age group (>65 years) for the accurate application of our kit (Fig. 2a–f). The demographic data based on age (1^st^–3^rd^ tertile) are shown in Supplementary Table 1. Next, we performed partial correlation analyses to identify the correlations between our *QPLEX*^*TM*^ biomarkers and cerebral amyloid deposition in different brain regions (four ROIs) (Fig. 3a-d). ROC curve analysis followed by logistic regression analysis was successively performed to identify the discrimination powers of our *QPLEX*^*TM*^ biomarkers in the 3^rd^ tertile (>65 years) group (Figs. 4a-d and 5a-c). Finally, we attempted to maximize the efficiency of our biomarkers by combining the *QPLEX*^*TM*^ biomarkers and plasma Aβ 1–42 (Fig. 5d, e). We achieved an area under the curve (AUC) of 0.921 with 95.7% sensitivity and 67.3% specificity. The experiments and analyses shown in Fig. 1b are described in greater detail below.

**Table 1:** Demographic data of the participants (*n* = 221).

Characteristics (*n*)	CN^−^ (185)	CN^+^ (36)	*P* value
Sex, M/F	80/105	20/16	0.1755^b^
Age, years, mean ± SEM	55.89 ± 1.2	74.47 ± 1.0	<0.0001^a^
Education, mean ± SEM	12.82 ± 0.3	12.08 ± 0.8	0.3846
MMSE raw score, mean ± SEM	27.78 ± 0.2	27.17 ± 0.4	0.1346
MMSE z score, mean ± SEM	0.48 ± 0.1	0.44 ± 0.1	0.7873
CDR (n)	0 (185)	0 (36)	–
ApoE4 positivity, ε4 + /N (%)	40/185 (22%)	12/36 (33%)	0.1305^b^
PiB (SUVR), mean ± SEM	1.09 ± 0.004	1.62 ± 0.05	<0.0001^a^

*CN* Cognitively normal, *PiB* Pittsburgh compound B, *− or +* PiB positivity, *SEM* standard error of the mean, *n* number of participants, *MMSE* Mini-Mental State Examination, *MMSE z score* revised value of the MMSE score with consideration for age, sex, and education level, *CDR* Clinical Dementia Rating, *ApoE* Apolipoprotein E, *SUVR* Standardized uptake value ratio, *N* total number of participants.

^a^significance by *t* test.

^b^significance by chi-square test.

### Age-dependent characterization of QPLEX^TM^ biomarkers without any effects of AD pathology

First, we tested the effects of age and sex, which are generally known as confounders of AD, on our biomarkers (Fig. 2a-c). Notably, we only used CN^−^ samples for Fig. 2a-c to exclude the effects of AD pathology on the results of testing the confounders (age and sex). Interestingly, our monotone regression spline analysis revealed three different curve patterns of biomarkers based on age (Fig. 2a). The cutoff dividing lines (42 and 65 years old) separating age groups were determined by the vertices of each biomarker curve (POSTN and ACE showed the first vertex at 42 years old; POSTN, ACE, and LGALS3BP showed the second vertex at 65 years old; Aβ1–40 showed unclear vertices). Based on this cutoff criterion, we defined three age groups as the 1st, 2nd, and 3rd tertiles. Interestingly, the second cutoff point (65 years old) exactly matched the standard age for late-onset AD (Fig. 2a)^31^. Next, we identified no differences in biomarkers between males and females (Fig. 2c). Furthermore, all of the biomarkers showed significant correlations between them (Fig. 2b) but with low values of the variance inflation factors (<2), which indicated that they could suitably be used as variables for logistic regression. Thus, based on these results, we concluded that biomarker analyses should be specified for the 3rd tertile age group (>65 years) for a more accurate application of our kit.

### Age-dependent approaches for identifying the correlation between QPLEX^TM^ biomarkers and cerebral amyloid deposition

Since the *QPLEX*^*TM*^ biomarkers showed different patterns among the age groups, we investigated the relationships between the biomarkers and cerebral amyloid deposition (SUVR) in each age group (1st tertile–3rd tertile) (Fig. 2d–f). The correlations between cerebral amyloid deposition and the *QPLEX*^*TM*^ biomarkers were not significant in the 1st and 2nd tertile groups. In the 3rd tertile group, we confirmed that POSTN and LGALS3BP levels in blood were significantly correlated with cerebral amyloid deposition (Fig. 2f). Thus, we reconfirmed the need to focus on the 3rd tertile group and further investigated whether the *QPLEX*^*TM*^ biomarkers could be utilized to discriminate PiB-PET positivity within this group. Moreover, the 1st and 2nd tertile groups were not appropriate for this analysis because they had too few PiB^+^ participants (Supplementary Table 1). We performed ANCOVA and partial correlation analysis once again for all ages with the correction for covariates (age and sex) to compensate for the age-dependent differences in biomarker levels (Supplementary Fig. 1a-d and Supplementary Table 2) and obtained significant results. However, since the ratio between CN^−^ and CN^+^ was relatively asymmetric (CN^−^, 185; CN^+^, 36; Table 1), we did not perform ROC curve analyses for all age groups.

### Relationship between the QPLEX^TM^ biomarkers and cerebral amyloid deposition in the 3^rd^ tertile group (>65 years)

Since our current criteria for PiB-PET positivity were based on the four ROIs in the brain (FR, LP, LT, and PC-PRC), we investigated the detailed correlations between cerebral amyloid deposition (SUVR) and *QPLEX*^*TM*^ biomarkers in each of the brain regions (ROIs)^23^. We performed a partial correlation analysis in the 3^rd^ tertile group and revealed that each biomarker showed a similar tendency across all brain regions (Fig. 3a-d). As expected, LGALS3BP and POSTN had higher correlations than ACE or Aβ1–40. Interestingly, the PC-PRC ROI showed the highest associations with *QPLEX*^*TM*^ biomarkers, including Aβ1–40 (Fig. 3d). Although there were no obvious differences between the brain ROIs (in terms of correlations), we decided to maintain our current criteria (at least 1 out of 4 SUVR ≥ 1.4, positive, CN^+^; all SUVR < 1.4, negative, CN^−^). Our participants were all CN individuals, and there have been many reports that more conservative thresholds are needed to detect preclinical AD among CN individuals^32^. We believed that if we used only the global (SUVR ≥ 1.4) criteria for PiB-PET positivity, we might have missed some patients in whom AD progression had already commenced.

Next, we performed a comparative analysis between CN^−^ and CN^+^ individuals in the 3^rd^ tertile group. Although the biomarkers did not show any differences between the sexes (Fig. 2c), we included this variable as a covariate because it is a well-known confounder of AD diagnosis^33^. Interestingly, two biomarkers, POSTN and LGALS3BP, showed significant differences between CN^−^ and CN^+^ individuals (Fig. 4a–d, left). The overall level of POSTN in blood was significantly higher in individuals with cerebral amyloid deposition above the threshold, whereas LGALS3BP showed the opposite tendency. The partial correlation analysis corrected for age and sex also revealed a significant association between blood biomarker levels and cerebral amyloid deposition (Fig. 4a-d, right). POSTN was positively correlated with cerebral amyloid deposition (partial correlation coefficient *R* = 0.2186, *P* = 0.0333), whereas LGALS3BP was negatively correlated with cerebral amyloid deposition (partial correlation coefficient *R* = −0.2626, *P* = 0.0094). When all the age groups were combined, all the *QPLEX*^*TM*^ biomarkers except for ACE showed significant correlations, as shown in Supplementary Fig. 1a-d. Furthermore, the ANCOVA results showed that all CN^+^ groups had significantly different levels of Aβ1–40, POSTN, and LGALS3BP than all CN^−^ groups (Supplementary Table 2).

### Discriminative ability of the QPLEX^TM^ Alz plus assay for CN^−^ vs. CN^+^ in the 3^rd^ tertile group (>65 years)

Although Aβ1–40 and ACE did not show significant results similar to the previous analyses (Figs. 3a-d and 4a-d; Supplementary Table 3), we decided to include Aβ1–40 and ACE in our biomarker panel because a multiple regression analysis including all CN groups (the dependent variable ‘cerebral amyloid deposition’ was included as one of the continuous variables) revealed that all the *QPLEX*^*TM*^ biomarkers, including Aβ1–40 and ACE, showed a significant correlation with cerebral amyloid deposition (SUVR) in every combination of variables (Table 2). We believe that these results indicate the possibility of potential associations for the logistic model with cerebral amyloid deposition. We performed logistic regression and ROC curve analysis to identify the discriminative ability of our *QPLEX*^*TM*^ Alz plus assay in the 3^rd^ tertile group (Fig. 5a). When the three ROC curves were compared with each other, we found that adding *QPLEX*^*TM*^ biomarkers dramatically increased the AUC (0.622 to 0.878, curve I vs. curve III; 0.684 to 0.878, curve II vs. curve III) (Fig. 5b). Each graph revealed high sensitivity and specificity (curve I, 82.4% sensitivity and 42.9% specificity; curve II, 52.9% sensitivity and 82.5% specificity; curve III, 69.7% sensitivity and 98.4% specificity) (Fig. 5c).

**Table 2:** Multiple regression analysis on cognitively normal individuals.

QPLEX^TM^ markers							
Dependent Y						Cerebral amyloid deposition (SUVR)	
Sample size						215	
Coefficient of determination R^2^						0.1329	
R^2^-adjusted						0.1163	
Multiple correlation coefficient						0.3645	
Residual standard deviation						0.2210	
Ind. variables	Coefficient	Std. Error	*t*	*P*	*r*_partial_	*r*_semipartial_	VIF
(Constant)	1.1778						
Aβ1–40	0.0009446	0.0003278	2.881	0.0044	0.1950	0.1851	1.131
LGALS3BP	−0.00002973	0.000008228	−3.613	0.0004	−0.2419	0.2322	1.211
ACE	−0.0004304	0.0001824	−2.359	0.0192	−0.1607	0.1516	1.465
POSTN	0.006219	0.001873	3.320	0.0011	0.2233	0.2134	1.587
**QPLEX**^**TM**^ **markers** **+** **Sex** **+** **Age**							
Dependent Y						Cerebral amyloid deposition (SUVR)	
Sample size						215	
Coefficient of determination R^2^						0.2613	
R^2^-adjusted						0.2400	
Multiple correlation coefficient						0.5112	
Residual standard deviation						0.2049	
Ind. variables	Coefficient	Std. Error	*t*	*P*	r_partial_	r_semipartial_	VIF
(Constant)	0.9098						
Aβ1–40	0.0006936	0.0003076	2.255	0.0252	0.1545	0.1344	1.158
LGALS3BP	−0.00002887	0.000007703	−3.747	0.0002	−0.2515	0.2233	1.234
ACE	−0.0003556	0.0001698	−2.094	0.0375	−0.1437	0.1248	1.476
POSTN	0.006376	0.001738	3.669	0.0003	0.2465	0.2186	1.588
Sex	−0.02188	0.02842	−0.770	0.4421	−0.0533	0.0459	1.024
Age	0.005013	0.0008386	5.977	<0.0001	0.3829	0.3562	1.026
**QPLEX**^**TM**^ **markers** **+** **Sex** **+** **Age** **+** **ApoE genotype**							
Dependent Y						Cerebral amyloid deposition (SUVR)	
Sample size						215	
Coefficient of determination R^2^						0.2810	
R^2^-adjusted						0.2567	
Multiple correlation coefficient						0.5301	
Residual standard deviation						0.2027	
Ind. variables	Coefficient	Std. Error	*t*	*P*	r_partial_	r_semipartial_	VIF
(Constant)	0.8908						
Aβ1–40	0.0006864	0.0003043	2.256	0.0251	0.1549	0.1329	1.158
LGALS3BP	−0.00002854	0.000007619	−3.746	0.0002	−0.2519	0.2207	1.234
ACE	−0.0003705	0.0001680	−2.205	0.0286	−0.1515	0.1299	1.478
POSTN	0.006314	0.001719	3.674	0.0003	0.2474	0.2165	1.589
Sex	−0.01911	0.02813	−0.679	0.4977	−0.0472	0.0400	1.025
Age	0.005049	0.0008295	6.087	<0.0001	0.3896	0.3587	1.027
ApoE	0.07754	0.03259	2.379	0.0183	0.1632	0.1402	1.006

*SUVR* standardized uptake value ratio, *Ind.* independent, *VIF* variance inflation factor, *Aβ1–40* beta-amyloid 1–40, *LGALS3BP* galectin-3 binding protein, *ACE* angiotensin-converting enzyme, *POSTN* periostin, *ApoE* apolipoprotein E.

### Maximization of the discrimination power for CN^−^ vs. CN^+^ by adding plasma Aβ1–42 as a variable in the 3rd tertile group

Since our *QPLEX*^*TM*^ Alz plus assay kit did not include plasma Aβ1–42, we conducted a further logistic regression analysis to maximize our discrimination power for CN^−^ vs. CN^+^ in the 3^rd^ tertile group (Fig. 5d-e). Due to limited resources, we only performed a bioplex assay using plasma samples from 75 participants of the 97 participants in the 3^rd^ tertile group. Since this assay is also a multiplex platform, we could obtain both plasma Aβ1–42 and Aβ1–40 values. Although the plasma Aβ1–42/1–40 ratio is a suitable biomarker for AD, our *QPLEX*^*TM*^ Alz plus assay kit showed a significantly higher AUC value (0.909) than that of the plasma Aβ1–42/1–40 ratio (Fig. 5e). Moreover, when we combined these variables (four *QPLEX*^*TM*^ biomarkers + plasma Aβ1–42; plasma Aβ1–40 from the bioplex assay could not be used as a variable due to statistical redundancy) to maximize the discrimination power for CN^−^ vs. CN^+^, the AUC reached 0.921 with 95.7% sensitivity and 67.3% specificity. These results likely suggest that our *QPLEX*^*TM*^ Alz plus assay can be used for prescreening amyloid deposition in the brain even when there are no apparent symptoms of cognitive disorders. Given these findings, the future development of the 2nd generation of our kit with plasma Aβ1–42 is highly encouraged.

## Discussion

Similar to the treatment of most diseases, including cancer and cardiac disease, AD has a better chance of being treated optimally before the severe progression of cognitive impairment, and the optimal strategy involves early diagnosis during the initial stage of AD pathogenesis^34^. Preclinical AD is a disease stage that initiates brain pathology and silent symptoms^34^. The identification of preclinical AD can be performed by assessing cerebrospinal fluid Aβ, tau, and p-tau using functional PET and MR imaging^35^. However, these modern imaging techniques are difficult to access due to their high cost and invasiveness^36,37^. Easily accessible and efficient blood-based biomarkers for diagnosis are necessary for the early detection and prevention of disease progression.

Previously, we identified blood biomarkers that can differentiate those with from those without cerebral amyloid deposition and suggested the possible relevance of these biomarkers in AD diagnosis^11^. Based on these blood biomarkers, the *QPLEX*^*TM*^ Alz plus assay kit was developed, and the efficiency of its diagnostic performance was assessed by clinically applying it to a large population cohort >55 years of age involving CN, MCI, and AD groups^21^. In the present study, we focused on *QPLEX*^*TM*^ biomarkers in CN individuals across a broad age range, including CN individuals <55 years of age.

First, we performed a monotone regression spline analysis using CN individuals, including those <55 years old, in which the participants’ age acted as a proxy for time (Fig. 2a). Previous studies have suggested that the levels of several blood biomarkers show different patterns based on age or sex in healthy individuals^38^. From this monotone regression spline analysis, we observed a general trend of changes in each *QPLEX*^*TM*^ blood biomarker based on age in the absence of AD pathology. With the CN status of participants confirmed based on the PiB-PET and Clinical Dementia Rating, we indirectly observed the manner in which our biomarkers underwent overall systemic changes under normal physiological conditions. Interestingly, the vertices of the monotone regression spline curve segmented the population into three age groups with distinct trends in the *QPLEX*^*TM*^ biomarkers. Further correlation analyses focusing on individual age groups revealed a significant correlation between cerebral amyloid deposition and biomarker levels in the 3rd tertile group (Fig. 2f). These results, coupled with the fact that 65 years is a clinical standard in sporadic AD and individuals in this age group are the most vulnerable to dementia, led us to focus mainly on the 3rd tertile group. Since the monotone spline curve in Fig. 2a was based on the ‘no cerebral amyloid deposition group’, the fluctuations in the curve were relatively minor compared to the range of change related to the progression of AD pathology. The aim of the monotone spline curve was to narrow down the subject of interest to the 3rd tertile group, which showed a consistent trend that was aligned with their age. The stable features in the 3rd tertile group were suitable for our biomarker analysis and needed to be specifically analyzed.

The abnormal atrophy and accumulation pattern of cerebral amyloid that occurs in specific brain regions is tightly associated with AD progression and can be utilized for diagnosis^39,40^. To investigate the correlation between biomarkers and pathology in detail, we performed a partial correlation analysis between each *QPLEX*^*TM*^ biomarker level and amyloid deposition in four different brain regions: PC-PRC, LP, FR, LT (Fig. 3a-d). Interestingly, LGALS3BP showed the most significant correlation, followed in descending order by POSTN, Aβ1–40, and ACE (Fig. 3a-d). This finding was fairly consistent with the results from a previous report that included CN, MCI, and AD groups^21^. The prior results from Park et al. and the results in Fig. 4a-d in this study demonstrate the most significant association of LGALS3BP, followed by POSTN with cerebral amyloid deposition in overall SUVR, suggesting that LGALS3BP and POSTN are valid blood biomarkers in various stages of disease progression. Aβ1–40 was most closely associated with the PC-PRC among the brain regions, which might be due to stage-specific vulnerability of the PC-PRC during AD pathogenesis. The PC-PRC is involved in the early stage of AD development^41–43^ and acts as one of the earliest regions of amyloid accumulation^40,44–47^. It has been implicated in beta-amyloid-related hypermetabolism in the early disease stage, showing abnormally increased amyloid accumulation^48^. From this point of view, we can speculate that the level of brain amyloid in the PC-PRC is better represented in the plasma because it is the predominant brain region that accumulates amyloid at the earliest stage. The PC-PRC also showed the greatest significance for LGALS3BP and the second greatest for POSTN (Fig. 3a-d), affirming that the region is implicated in early brain amyloid accumulation.

Multiple previous reports have demonstrated that plasma Aβ1−42 and tau levels are efficient biomarkers for cerebral amyloid deposition^12,49–51^. Moreover, the analysis of plasma Aβ42/40 ratios has identified cerebral amyloid deposition not only in prodromal AD but also in preclinical AD^52,53^. Thus, we performed an additional quantification of plasma Aβ1−42 levels and compared the diagnostic power among the plasma Aβ42/40 ratio, *QPLEX*^*TM*^ biomarkers, and *QPLEX*^*TM*^ biomarkers combined with the plasma Aβ42/40 ratio (Fig. 5d, e). The *QPLEX*^*TM*^ Alz plus assay kit showed a significantly higher AUC value than the plasma Aβ42/40 ratio, and the highest AUC value was observed with *QPLEX*^*TM*^ combined with plasma Aβ 1-42 (AUC: 0.921). Due to limited resources, different numbers of individuals were included in the ROC analyses that included Aβ1–42, that is, Fig. 5d, e, compared to the analysis in Fig. 5a–c. Although it was not possible to directly compare the ROC results between Fig. 5a–c and Fig. 5d, e due to the difference in numbers, when we compared the AUC values with the similar range of the sensitivity (81–82%) between the two models, the inclusion of Aβ1–42 with *QPLEX*^*TM*^ increased the efficiency of the diagnosis compared to *QPLEX*^*TM*^ alone from 0.878 (81.82% sensitivity, 67.21% specificity) to 0.921 (82.61% sensitivity, 78.85% specificity). This result proves the efficiency of the *QPLEX*^*TM*^ Alz plus assay kit, as well as the synergistic effect of the combinational use of *QPLEX*^*TM*^ Alz plus assay kit with well-known biomarkers such as plasma Aβ 1-42.

In this study, Aβ1−42 was quantified with xMAP, which is considered a highly available and cost-effective method. Recently developed detection methods, such as SIMOA, can offer a lower detection limit by reducing fluorescence signal diffusion, and IP-MS is beneficial in that trace amounts of plasma Aβ can first be enriched by immunoprecipitation and then subsequently analyzed with mass spectrometry. However, this fastidious procedure is difficult to perform in the clinic or hospital for diagnostic purposes. The difficulty in quantifying plasma Aβ1−42 levels due to aggregation tendency and low concentration in blood makes it difficult to include this biomarker in the diagnostic kit, which is a limitation of our study. We look forward to incorporating this biomarker into the panel in the near future, as its inclusion showed higher performance.

Many assays for AD blood biomarkers have been validated to date. Fragments of beta-amyloid or the ratio between isoforms are extensively being investigated^54^. Using IP-MS, Aβ1-42 itself or ratios such as APP669-711/Aβ1-42 and Aβ1-40/Aβ1-42 can be used to discriminate patients with AD from controls^55^. Peripheral tau protein phosphorylation at particular sites indicates brain pathology and can be used to track CNS changes in those with AD^56^. p-tau181, which is increased in preclinical AD^57^, is a scalable marker for predicting and monitoring neurodegeneration in an AD-specific manner^58^. Plasma p-tau217 is also useful; some studies have shown that it has higher accuracy than plasma p-tau181 in detecting abnormal CNS tau metabolism^56,59^. Recently, plasma p-tau231 was validated as it was correlated with CSF p-tau231 and was useful for predicting incipient AD pathology^60^.

Biomarkers in the *QPLEX*^*TM*^ panel are advantageous in that these novel biomarkers have not been extensively discussed in the AD context, and they provide hints regarding the underlying mechanisms of preclinical AD. For example, changes in LGALS3BP and POSTN indicate that the peripheral immune response may have already been altered in preclinical AD^17,19,20^. As AD is a multifactorial disease, different biomarkers reflect different aspects of AD. While canonical plasma markers such as phosphorylated tau and beta-amyloid fragments, including our plasma Aβ1−40, aim to detect direct products from the brain, the biomarkers in our panel, such as LGALS3BP, ACE, and POSTN, may reflect secondary effects in the periphery that are primarily caused by AD brain pathology. When our biomarkers are used in conjunction with the panel of previously known blood biomarkers, they can help separate and stratify the patients by identifying the disease subtypes^8,10,61^.

The kit is practical for use in cognitively normal individuals in various ways. Tests using this kit could be included in regular check-ups or routine physical examinations of elderly individuals, especially those over 65 years old, to detect AD pathogenesis in the early stage. On the other hand, this kit could be the first choice for cognitively normal individuals who visit the doctor’s office due to concerns of their AD probability based on family history or ApoE genotype. Additionally, the kit can satisfy the needs of cognitively normal individuals who wish to take precautionary measures without costly brain imaging and be used to screen individuals eligible for clinical trials.

## Supplementary information

Supplementary Information
