# Supplementary material for: Application of QPLEXTM biomarkers in cognitively normal individuals across a broad age range and diverse regions with cerebral amyloid deposition
Source: Exp Mol Med. 2022 Jan 20;54(1):61–71. doi: 10.1038/s12276-021-00719-3 (PMC8814000; doi:10.1038/s12276-021-00719-3)
Supplement: Supplementary file 1 — Supplementary Information [file 12276_2021_719_MOESM1_ESM.pdf]

**Supplementary Information**

# Application of *QPLEX*$^{TM}$ biomarkers in cognitively normal individuals across a broad age range and diverse regions with cerebral amyloid deposition

Lee *et al.*

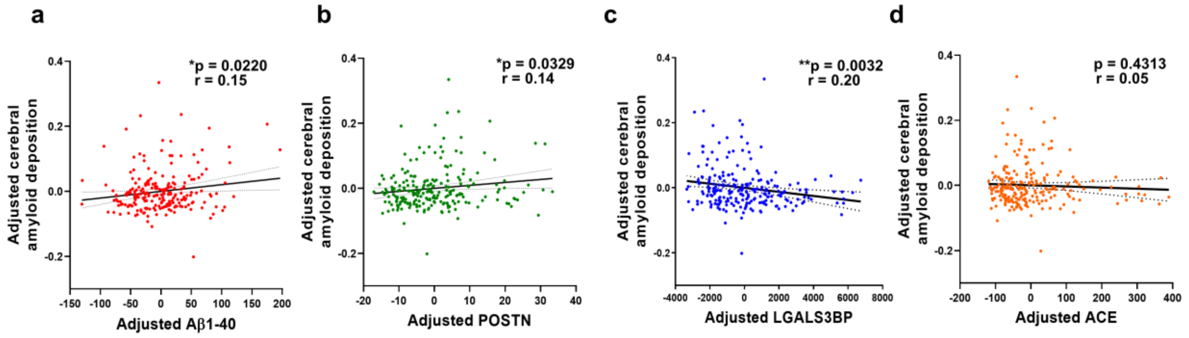


**Supplementary Fig. 1 The QPLEX™ biomarkers and cerebral amyloid deposition for all CN groups (including all age-groups) a-d** Each graph on the left shows the correlation of QPLEX™ biomarker levels with cerebral amyloid deposition for all CN groups. All graphs show the results from the partial correlation analysis with correction for age and sex. P-value cut-off, $*p < 0.05$, $**p < 0.01$, $***p < 0.001$, and $****p < 0.0001$. P, partial correlation P-value; R, partial correlation coefficient. Dotted lines indicate 95% confidence intervals; CN, cognitively normal

**Supplementary Table 1. Demographic data of the participants (based on age-groups)**

| Characteristics (n) | 1st tertile (47) | 2nd tertile (77) | 3rd tertile (97) | P-value |
|---|---|---|---|---|
| **Sex, M/F** | 24/23 | 32/45 | 44/53 | 0.5871† |
| **Age, years, mean ± SEM (range)** | 32.04 ± 0.9 (21 to 42) | 56.25 ± 0.7 (43 to 65) | 74.06 ± 0.5 (66 to 87) | < 0.0001* |
| **Education, mean ± SEM** | 15.19 ± 0.3 | 12.83 ± 0.5 | 11.39 ± 0.5 | < 0.0001* |
| **MMSE raw score, mean ± SEM** | 29.13 ± 0.1 | 28.12 ± 0.2 | 26.64 ± 0.2 | < 0.0001* |
| **MMSE z score, mean ± SEM** | 0.80 ± 0.1 | 0.55 ± 0.1 | 0.26 ± 0.1 | < 0.0001* |
| **CDR (n)** | 0 (47) | 0 (77) | 0 (97) | 1 |
| **ApoE4 positivity, ε4+/N (%)** | 13/47 | 15/77 (%) | 24/97 | 0.5416† |
| **PiB (SUVR), mean ± SEM** | 1.08 ± 0.01 | 1.09 ± 0.01 | 1.29 ± 0.03 | < 0.0001* |
| **PiB positivity (- / +), n** | 47/0 | 75/2 | 63/34 | < 0.0001† |

CN, cognitively normal; PiB, Pittsburgh compound B; - or +, PiB positivity; SEM, standard error of the mean; n, number of participants; MMSE, Mini-Mental State Examination; MMSE z score, a revised value of the MMSE score considering age, sex, and education level; CDR, Clinical Dementia Rating; ApoE, Apolipoprotein E; SUVR, standardized uptake value ratio; N, total number of participants.

- *, significance by *t*-test; †, significance by chi-squared test.

**Supplementary Table 2. Comparison of QPLEX™ biomarker levels between CN- and CN+ (including all age-groups) with correction for age and sex**

| Factors | Mean difference | Std. Error | P[a] | 95% CI[a] |
|---|---|---|---|---|
| **Aβ1-40** | -22.3647 | 9.7673 | 0.0230 | -41.6166 to -3.1128 |
| **POSTN** | -5.1039 | 2.0547 | 0.0138 | -9.1539 to -1.0540 |
| **LGALS3BP** | 1801.1584 | 390.4209 | <0.0001 | 1031.6559 to 2570.6610 |
| **ACE** | 10.0631 | 20.1814 | 0.6186 | -29.7177 to 49.8439 |

[a] Bonferroni corrected

***Abbreviations:** DF, degrees of freedom; F, F statistic; P, significance level; Std. Error, standard error; CI, confidence interval; PiB-PET, Pittsburgh compound B positron emission tomography; Aβ1-40, amyloid beta 1-40; POSTN, periostin; LGALS3BP, galectin-3 binding protein; ACE, angiotensin-converting enzyme.

**Supplementary Table 3. Multiple regression analysis on the 3rd tertile group (>65 years)**

| QPLEX™ markers | | | | | | | |
|---|---|---|---|---|---|---|---|
| **Dependent Y** | | | | | | | **Cerebral amyloid deposition (SUVR)** |
| **Sample size** | | | | | | | 94 |
| **Coefficient of determination $R^2$** | | | | | | | 0.2437 |
| **$R^2$- adjusted** | | | | | | | 0.2097 |
| **Multiple correlation coefficient** | | | | | | | 0.4937 |
| **Residual standard deviation** | | | | | | | 0.2791 |
| **Ind. variables** | **Coefficient** | **Std. Error** | **t** | **P** | **$r_{partial}$** | **$r_{semipartial}$** | **VIF** |
| **(Constant)** | 1.3670 | | | | | | |
| **Aβ1-40** | 0.0008808 | 0.0005743 | 1.534 | 0.1286 | 0.1605 | 0.1414 | 1.165 |
| **LGALS3BP** | -0.00005646 | 0.00001641 | -3.440 | 0.0009 | -0.3426 | 0.3171 | 1.214 |
| **ACE** | -0.0008850 | 0.0004007 | -2.209 | 0.0298 | -0.2280 | 0.2036 | 1.506 |
| **POSTN** | 0.01381 | 0.003477 | 3.973 | 0.0001 | 0.3882 | 0.3663 | 1.492 |
| **QPLEX™ markers + Sex + Age** | | | | | | | |
| **Dependent Y** | | | | | | | **Cerebral amyloid deposition (SUVR)** |
| **Sample size** | | | | | | | 94 |
| **Coefficient of determination $R^2$** | | | | | | | 0.2468 |
| **$R^2$- adjusted** | | | | | | | 0.1948 |
| **Multiple correlation coefficient** | | | | | | | 0.4968 |
| **Residual standard deviation** | | | | | | | 0.2817 |
| **Ind. variables** | **Coefficient** | **Std. Error** | **t** | **P** | **$r_{partial}$** | **$r_{semipartial}$** | **VIF** |
| **(Constant)** | 1.2060 | | | | | | |
| **Aβ1-40** | 0.0009375 | 0.0005877 | 1.595 | 0.1143 | 0.1686 | 0.1484 | 1.198 |
| **LGALS3BP** | -0.00005411 | 0.00001703 | -3.178 | 0.0021 | -0.3225 | 0.2957 | 1.282 |
| **ACE** | -0.0008844 | 0.0004051 | -2.183 | 0.0317 | -0.2279 | 0.2031 | 1.511 |
| **POSTN** | 0.01317 | 0.003694 | 3.565 | 0.0006 | 0.3570 | 0.3317 | 1.653 |
| **Sex** | -0.02716 | 0.06029 | -0.450 | 0.6535 | -0.04824 | 0.04191 | 1.064 |
| **Age** | 0.002239 | 0.005912 | 0.379 | 0.7058 | 0.04057 | 0.03523 | 1.108 |
| **QPLEX™ markers + Sex + Age + ApoE genotype** | | | | | | | |
| **Dependent Y** | | | | | | | **Cerebral amyloid deposition (SUVR)** |
| **Sample size** | | | | | | | 94 |
| **Coefficient of determination $R^2$** | | | | | | | 0.2778 |
| **$R^2$- adjusted** | | | | | | | 0.2190 |
| **Multiple correlation coefficient** | | | | | | | 0.5270 |
| **Residual standard deviation** | | | | | | | 0.2775 |
| **Ind. variables** | **Coefficient** | **Std. Error** | **t** | **P** | **$r_{partial}$** | **$r_{semipartial}$** | **VIF** |
| **(Constant)** | 1.3271 | | | | | | |
| **Aβ1-40** | 0.0008562 | 0.0005804 | 1.475 | 0.1438 | 0.1571 | 0.1352 | 1.204 |
| **LGALS3BP** | -0.00005650 | 0.00001682 | -3.360 | 0.0012 | -0.3406 | 0.3079 | 1.289 |
| **ACE** | -0.0007833 | 0.0004025 | -1.946 | 0.0549 | -0.2054 | 0.1784 | 1.537 |
| **POSTN** | 0.01257 | 0.003652 | 3.442 | 0.0009 | 0.3480 | 0.3154 | 1.665 |
| **Sex** | -0.02361 | 0.05941 | -0.397 | 0.6921 | -0.04281 | 0.03642 | 1.065 |
| **Age** | 0.0004404 | 0.005897 | 0.0747 | 0.9406 | 0.008053 | 0.006844 | 1.137 |
| **ApoE** | 0.1317 | 0.06859 | 1.921 | 0.0581 | 0.2028 | 0.1760 | 1.061 |

***Abbreviations:** SUVR, standardized uptake value ratio; Ind., independent; VIF, variance inflation factor; Aβ1-40, amyloid beta 1-40; LGALS3BP, galectin-3 binding protein; ACE, angiotensin-converting enzyme; POSTN, periostin; ApoE, apolipoprotein E.
